# BARRIERS AND FACILITATORS TO PHYSICAL ACTIVITY OF OLDER ADULTS IN KOREAN NURSING HOMES: A QUALITATIVE STUDY

**DOI:** 10.1093/geroni/igae098.3808

**Published:** 2024-12-31

**Authors:** Young Ko, Seon Kyung Yun, Geun Sil Yook, Seo Young Kim

**Affiliations:** Gachon University, Incheon, Republic of Korea; Saekyung University, Yeongwol, Kangwon-do, Republic of Korea; Gachon University, Incheon, Republic of Korea; Gachon University, Incheon, Republic of Korea

## Abstract

Various factors can either reduce or improve the physical activity level of older people living in nursing homes. Although it is critical to manage the risk of inactivity, only a few studies have investigated the multifaceted factors associated with physical activity among nursing home residents. The purpose of this study was to investigate directors’ and care workers’ perceptions of barriers and facilitators to physical activity for older adults in Korean nursing homes. This was a qualitative descriptive study. We collected data from January 13 to February 14, 2023. We used thematic analyses to analyze data from 4 directors and 22 long-term care workers in nursing homes in South Korea. We extracted 4 themes and 11 sub-themes reflecting the factors related to older adults in nursing homes: (a) “safety is more important than activity,” (b) “encourage activities tailored to their preference,” (c) “activities in harmony,” and (d) “meaning of living at a nursing home.” Individual, interpersonal, environmental, and policy factors influence older adults’ physical activity in nursing homes. These factors guide policymakers, healthcare providers, and care workers in developing and implementing activity planning to help improve recipients’ physical activity level in nursing homes. This research was supported by the Basic Science Research Program through the National Research Foundation of Korea (NRF) funded by the Korean government (MSIT) (No. 2022R1F1A1074082).

